# Well-Being Among Aging Women: Observations From the Women’s Health Initiative Study (WHI)

**DOI:** 10.1093/geroni/igab046.1817

**Published:** 2021-12-17

**Authors:** Barbara Cochrane, Barbara Cochrane

## Abstract

Previous efforts to assess well-being in relation to health have relied on descriptive analyses of hedonic or eudaemonic well-being indicators. Factor scores from principal components analysis offer a summary measure of well-being, but limited interpretability in epidemiologic analyses, e.g. estimated risk ratios. Use of latent class analysis to identify groups differing by levels of both hedonic and eudaemonic indicators preserves information about both dimensions while supporting interpretation of well-being effects on health indicators important for studying older women’s health. For this symposium, we report on analyses to: (1) Develop a multidimensional profile of well-being that would preserve the individual dimensions of well-being (hedonic, eudaemonic) using latent class analysis; and (2) Support epidemiologic analyses of well-being as both an outcome and a predictor of health outcomes, enhancing interpretability of levels of well-being across various dimensions. Data on well-being in over 80,000 older women in the Women’s Health Initiative Study were obtained in 2011-2012, along with their baseline demographic characteristics in 1993-1998. All-cause mortality included death from any cause occurring from 2012-2020. Four classes of well-being were identified: (1) lowest hedonic and eudaemonic well-being scores; (2) higher hedonic/lower eudaemonic scores; (3) lower hedonic/higher eudaemonic scores; and (4) highest hedonic and eudaemonic scores. Results will be presented in three papers addressing the development of the well-being profile; relationships of the profile with predictors of well-being; and the relationship of the profile to all-cause mortality. The discussion will focus on future research questions, such as likely factors mediating well-being and health outcomes.

